# Mechanism of charge accumulation of poly(heptazine imide) gel

**DOI:** 10.1038/s41598-021-97025-9

**Published:** 2021-09-08

**Authors:** Goichiro Seo, Yuki Saito, Miyu Nakamichi, Kyohei Nakano, Keisuke Tajima, Kaname Kanai

**Affiliations:** 1 Department of Physics, Faculty of Science and Technology, Tokyo University of Science, 2641 Yamazaki, Noda, Chiba 278-8510 Japan; 2 RIKEN Center for Emergent Matter Science (CEMS), 2-1 Hirosawa, Wako, Saitama 351-0198 Japan

**Keywords:** Photocatalysis, Optical materials, Surface spectroscopy

## Abstract

Photo-stimuli response in materials is a fascinating feature with many potential applications. A photoresponsive gel of poly(heptazine imide), PHI, termed PHIG, exhibits photochromism, photoconductivity, and photo-induced charge accumulation, and is generated using ionic liquids and PHI. Although there are several examples of ionic liquid gels that exhibit photochromism and photoconductivity, this is the first report of an ionic liquid gel that exhibits both these properties as well as charge accumulation. We conducted experimental and theoretical investigations to understand the mechanism of the photostimulus response of PHIG, especially charge accumulation. The proposed model explains both the mechanism of charge accumulation and dark photocatalysis by PHI and provides new concepts in the field of photofunctional materials.

## Introduction

Light causes changes in the chemical and electronic states of photo-responsive materials, thereby imparting functionality. Such materials have been studied for many years. Applications of light-induced functions include solar cells, photoconductors, photoinduced insulators, photochromism, photocatalysis, solidification and viscosity changes due to light irradiation, and light-induced magnetism^1–4^. Among them, both photochromism and photocatalysis are useful and have been now widely applied in society^5–7^. Photochromism is the reversible color change caused by light irradiation. Photocatalysis can generate hydrogen by decomposing water and organic materials by light irradiation. As is well known, TiO_2_ is typical photocatalysis and which has been used in a wide range of applications such like coating agents to decompose stains on exterior walls^6,7^. Photocatalysts in particular hold promise also in public health for antibacterial and antiviral applications.

**Figure 1:**
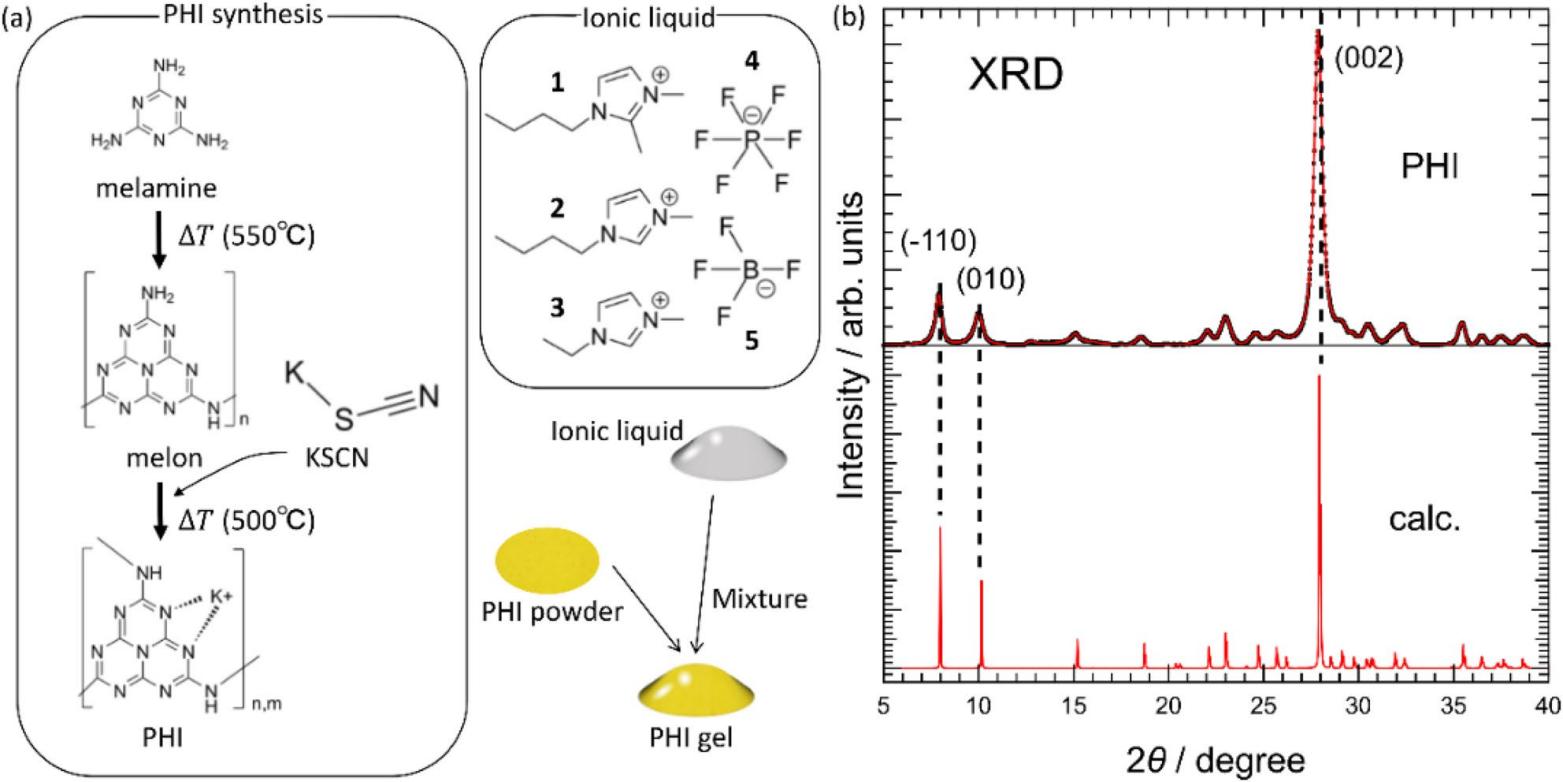
(**a**) Schematic scheme for the synthesis of PHI and PHIG. An ionic liquid is composed of a combination of one cation and one anion. The molecules labeled **1**, **2** and **3** are typical cations used in ionic liquids, Bmmim^+^, Bmim^+^ and Emim^+^, respectively. The molecules labeled **4** and **5** are typical anions used in ionic liquids, PF_6_^−^ and BF_4_^−^, respectively. (**b**) XRD profile of powdery PHI in the upper panel. The solid lines represent the fitting curves obtained by the least-squares method. The lower panel shows the calculated diffraction pattern of the model of K-PHI proposed in the literature^19^.

Graphitic carbon nitride has been actively studied as a photocatalyst for generating hydrogen through photocatalytic activity using the visible wavelengths of sunlight, whereas TiO_2_ requires ultraviolet light. Graphitic carbon nitride is a two-dimensional material composed only of carbon, nitrogen, and hydrogen. As typical examples, g-C_3_N_4_ and melon are a two-dimensional and quasi-one-dimensional polymer with a heptazine skeleton, respectively^8,9^, and poly(triazine imide) is a two-dimensional polymer with a triazine skeleton^10^. Poly(heptazine imide) (hereafter referred to as PHI) exhibits photocatalysis and was first reported by Savateev et al.^11^ (shown in Fig. 1a). PHI has attracted much attention recently because of its interesting functions: (1) reversible color change upon visible light irradiation, and (2) dark photocatalysis reported by Lau et al.^12^. Generally, in dark photocatalysis, the charges generated by light irradiation are stored inside the material and are consumed in the dark state, as first confirmed in the TiO_2_-WO_3_ system^13,14^. This photocatalytic function can significantly improve the efficiency of hydrogen collection as “day–night photocatalysis”. In the case of PHI, a model called “IDEAS” (illumination-driven electron accumulation in semiconductors) proposes that charges are accumulated in PHI by charge donation from an electron donor under light irradiation^15^. The functions (1) and (2) have been actually reported for PHI solutions doped with organic electron donors, and the IDEAS explains that they are derived from charge donation by the electron donors to PHI. However, there is currently no reported evidence that an electron donor actually donates charge to PHI upon light irradiation. Here, we show that the electron donation does not necessarily play an essential role in the charge accumulation in PHI because we demonstrate that the color change of PHI due to light irradiation occurs even without electron donors. We propose a new model to explain functions (1) and (2) that occur in PHI: (1) is caused by photochromism associated with structural changes of PHI, and (2) is due to charge accumulation by shallow traps generated in PHI by light irradiation. These two phenomena are different, but both are caused by changes in the electronic state of PHI triggered by light irradiation and are strongly correlated each other. Previously, the photochromism of PHI has been reported to occur only in solution under severely constrained conditions. In this study, we addressed this by making PHI into a gel. PHI gel (PHIG) can be obtained by mixing PHI and an appropriate ionic liquid, which has an extremely low vapor pressure and very high viscosity. The gel made by using ionic liquids were firstly reported by Fukushima et al. They reported that when mixed with imidazolium-based ionic liquid, pristine single wall carbon nanotube formed gels^16^. Here, we adopted their similar method to form gels of PHI. It has been reported that some gels made using ionic liquids showing photochromism have been previously reported^17,18^ but gels showing charge accumulation in this study are unique. Furthermore, gelation of PHI also has advantages in elucidating its chemical states because PHIG allows experiments to explore the properties of PHI, including experiments performed in a vacuum such as photoelectron spectroscopy. Here we studied the photochromism of PHI by the combination of UV–Vis absorption (UV–Vis), X-ray photoelectron spectroscopy (XPS), reflection spectroscopy, and calculations using density functional theory (DFT). In addition, the photocurrent of PHIG was measured to understand the mechanism of charge accumulation in PHI. Another advantage of using ionic liquids for PHIG is that they are transparent to visible light and do not interfere with the light absorption of PHI. Finally, we propose the “shallow trap charge accumulation” model as a new mechanism for dark photocatalysis by PHI. The findings in this work provide new guidelines for the development of compounds that exhibit dark photocatalysis.

## Experimental and methods

### Synthesis methods

PHI was obtained by the thermal treatment of the precursor melon. Melon was synthesized in a quartz tube placed in a commercial tube furnace (Koyo Thermo Systems Co., Ltd., KTF035N1) under a nitrogen atmosphere (purity: 99.99995%)^18^. All quartz tubes and boats were pre-annealed to remove contaminants, and NH_3_ and CS_2_ gas were removed using a fixed flow rate of N_2_ gas at 200 mL min^−1^ before each synthesis.

To synthesize melon, melamine (3.0 g, purity: 99.0% Wako Pure Chem., Ind., Ltd., 139-00945) was placed in the bottom of a quartz tube (Nichiden-Rika Glass Co., Ltd., P-18SM) and the tube was capped with aluminum foil with a central pinhole ~ 0.6 mm in diameter. The aluminum foil was fixed with tungsten wire and thermal polymerization was conducted in this semi-closed system. The melamine was heated to 550 °C at a heating rate of 1 °C min^−1^ and then held at 550 °C for 5 h. The sample was then cooled to room temperature at 2 °C min^−1^.

PHI was synthesized in a quartz tube as described in the literature^19^. Melon (0.3 g) and KSCN (0.15 g, purity: 98.0% Wako Pure Chem., Ind., Ltd., 164-04555) were mixed and placed in a combustion boat (NIKKATO CORPORATION). The boat was placed at the bottom of a quartz tube and the tube was capped with aluminum foil, as described above. The boat was rapidly heated to 400 °C at a heating rate of 30 °C min^−1^ and then held at 400 °C for 1 h. After this first heating, the mixture was thermally treated again by heating to 500 °C at a heating rate of 30 °C min^−1^, held at 500 °C for 30 min, then cooled to room temperature at a cooling rate of 2 °C min^−1^. The product was washed with acetone and water and separated by centrifugation. Finally, the obtained sample was dried in a desiccator to obtain PHI as a yellow powder.

As shown in Fig. 1a, PHIG was obtained by mixing PHI (15 mg) and ionic liquid (20 mL). All ionic liquid samples were purchased from Kanto Chemical Co. Inc., and were used without further purification. Ionic liquids with a fluoride anion were used to make PHIG: 1-butyl-3-methylimidazolium tetrafluoroborate ([Bmim]BF_4_), 1-butyl-3-methylimidazolium hexafluorophosphate ([Bmim]PF_6_), 1-butyl-4,5-dimethyl-imidazole tetrafluoroborate ([Bmmim]BF_4_), 1-butyl-3-methylimidazolium Bromide ([Bmim]Br) and 1-ethyl-3-methylimidazolium hexafluorophosphate ([Emim]PF_6_). PHIGs were successfully obtained using all ionic liquids.

### Analysis methods

All photochromism studies of PHI were performed using a white LED (HAYASHI-REPIC, LA-HDF 100NA) as a visible light source.

UV–Vis measurements (V-650 spectrometer, JASCO) of PHIG were performed on samples placed between two quartz plates to combine transmission and diffuse reflection methods. The time variation of specific wavelengths about the PHIG sample between the glass plates was measured using a spectrometer (BIM-6002A, Brolight) in a homemade dark box. The KBr pellet method was used to record FT-IR spectra of the powder samples. XRD analyses were carried out using a diffractometer (Ultima IV, Rigaku) with a Cu Kα radiation source (*λ* = 0.15496 nm).

Electric current measurements were performed on PHIG pasted on patterned ITO substrate using a source-measure unit (6487, Kethley) under constant voltage (0.5 V) generated by a DC power source (R6144, Advantest).

XPS measurements were performed on PHIG using a PHI 5000 Versa Probe (Ulvac-Phi) instrument with monochromatic Al Kα (*hν* = 1486.6 eV) radiation as the excitation source. The XPS spectra were analyzed with Voigt functions in the XPSPEAK41 software program (written by Raymund W. M. Kwok). PHIG[blue] was probed using a blue laser (*λ* = 473 nm, KYOCERA SOC, J010BS).

### Theoretical calculations

Energy band calculations were performed using density functional theory (DFT). Models of PHI[K-0], PHI[K-1], and PHI[K-3] were created using Material Studio software (Dassault Systèmes BIOVIA). The band calculations for PHI[K-0], PHI[K-1], and PHI[K-3] were obtained using GGA/PBE with the double numerical (DND) basis set in DMol3. Optical spectra were simulated using time-dependent DFT (TD-DFT) with ALDA. XRD profiles were calculated using the Powdery Diffraction Pattern package in the Visualization for Electronic and Structural Analysis program (VESTA), and the previously reported structure of K-PHI were used for the calculations^19^.

## Results and discussion

### Photochromism of poly(heptazine imide)

The XRD spectrum of the synthesized PHI is shown in the upper panel of Fig. 1b, and the lower panel shows the calculated diffraction pattern of the model of PHI proposed by Schlomberg et al.^19^*.* The calculated XRD spectrum well explains the experimental spectrum, including the (002), (− 110), and (010) diffractions. The FT-IR spectrum of PHI in Fig. S1 in “Supporting Information S1” shows the ν (CN) vibration of a CN heterocyclic structure such as melon, but also clearly shows ν (C≡N), indicating that PHI has a cyano-group instead of the amino group seen in the melon spectrum. Note that spectra of PHI is also different from Poly(triazine imide) especially 1500–1750 cm^−1^, caused by difference between heptazine and triazine ring^20^.

The XRD and FT-IR results are in good agreement with those of previous studies^19,21^, indicating that PHI was successfully synthesized.

Previous studies showed that PHI changes color in response to light^12^ but only when it is in a solvent, not as a powder, which has prevented research into applications of this material. Here, we succeeded in removing PHI from the solvent by forming a gel by mixing PHI with an ionic liquid. PHIG also changes color under irradiation with visible light, as in solution. It has been confirmed by UV–Vis measurements that there is no degradation of PHI due to gelation as shown in Fig. S2. Furthermore, because ionic liquids are very viscous and extremely low vapor pressure polar solvents, PHIG is stable even under ultra-high vacuum, allowing experiments in a vacuum, such as photoelectron spectroscopy. Such measurements provided many new insights into the properties of PHI including the charge accumulation and are presented below.

**Figure 2:**
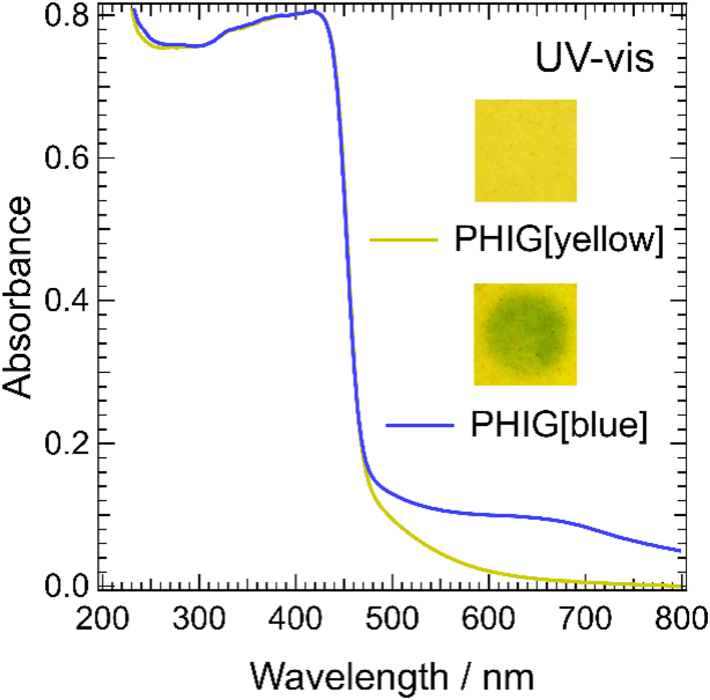
UV–Vis spectra of PHIG with the ionic liquid [Bmim]PF_6_. PHIG[yellow] means the sample before white-light irradiation, PHI[blue] means the sample after the irradiation for 30 min. Photos in the figure represent PHIG[yellow] and PHIG[blue].

Figure 2 shows the results of UV–Vis measurements of PHIG. PHIG[yellow] is the sample in yellow before white-light irradiation and PHIG[blue] is the sample in blue after white-light irradiation. Comparing PHIG[yellow] and PHIG[blue], irradiation with white light does not affect the spectrum below 470 nm but increases the intensity near 650 nm, indicating that the absorption wavelength range of PHIG is expanded by white-light irradiation. The reason why the change in the absorption spectrum seems to be limited compare to its appearance is that the entire sample did not change color and the light-irradiated area on the surface of PHIG is smaller than the probed area. Furthermore, with a short irradiation time, some yellow areas remain even on the surface of PHIG[blue]. PHIG[blue] shows a reversible change in which the color gradually returns to yellow after turning off the light at room temperature, indicating that it is a T-type photochromic substance with thermal decolorization. A similar color change by PHI was reported by Lau et al.^12^.

### Role of the electron donor in photochromism of PHI

**Figure 3:**
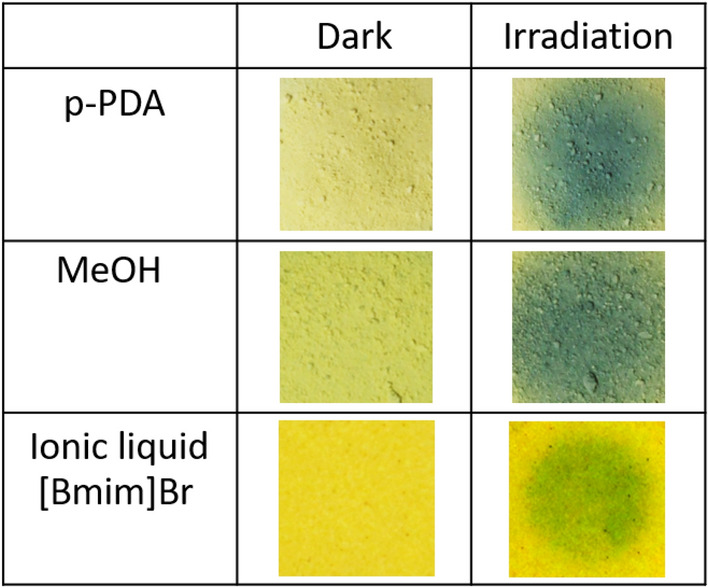
Photographs of PHI before and after white-light irradiation. The sample in the upper panel contains PHI and 0.05 mol/L of p-PDA as an electron donor dissolved in MeOH. The sample in the middle panel is PHI alone immersed in MeOH. The sample in the bottom panel contains PHIG which is composed by PHI and [Bmim]Br.

Previous studies on the photochromism of PHI considered that the supply of electrons from the electron donor to PHI causes new absorption^15^ in the visible wavelength range, resulting in the color change of PHI under irradiation. However, the experimental results given below cannot be explained by this mechanism. In the upper and middle panels of Fig. 3, PHI was immersed in MeOH and lower panel PHI was immersed in Ionic liquid.

In the upper panel, p-PDA (1,4-diaminobenzene), a typical organic electron donor, was dissolved together with PHI. In the lower panel, PHI alone was immersed in MeOH. Note that neither sample is a gel, allowing direct comparison with previous reports. The color of PHI with p-PDA and MeOH changes to blue upon light irradiation. The results obtained using other electron donors (4-MBA: 4-methylbenzyl alcohol and BA: benzylamine) are shown in Fig. S3a: PHI changes to blue by light irradiation regardless of the electron donor used. The IDEAS model for the color change in PHI assumes that the charge supplied to PHI by an electron donor is triggered by light irradiation^15^. However, the same color change was observed (lower panel) for PHI without an electron donor. Given that ionic liquid is unlikely to be an electron donor because of its large bandgap^22^, the results in Fig. 3 indicate that an electron donor is not necessarily required for PHI to change color when exposed to light. The same experiments were performed using other solvents and the results are shown in Fig. S3b. The photochromism of PHI occurs in certain solvents. There was an obvious color change of PHI in MeOH and glycerin, and a slight color change in H_2_O. A previous study^23^ reported that adding 4-MBA to PHI in H_2_O resulted in a color change in PHI, and this color change is due to 4-MBA because PHI immersed in H_2_O does not undergo a color change. However, this does not prove electron donation from 4-MBA to PHI. Figure S4 shows the electron spin resonance (ESR) results for a MeOH solution of PHI. According to previous studies^15,19^, when PHI receives an electron from the donor, electron radicals should be generated and a sharp peak should appear at 3350 Gauss in the ESR spectrum, but this was not observed for either sample, as shown in Fig. S4. This indicates that PHI does not have electron radicals even when mixed in solution with 4-MBA. A previous study reported that PHI[blue] has a strong ESR signal, which is different from our results. The synthesis methods that were used to prepare PHI in this work and the previous work were essentially identical. Furthermore, the XRD patterns and FT-IR spectra of the obtained PHI are very similar in this and previous work. However, in the previous study^15^, an ESR signal appeared even for PHI[yellow]. One possible origin for this discrepancy might be due to a small amount of impurity contained in PHI (due to differences in synthetic strategy and sample handling) that gave rise to ESR signals.

The IDEAS model previously suggested to explain the color change of PHI cannot explain the results of Fig. 3 and Fig. S4 because it assumes electron donation from the electron donor to PHI under light irradiation. We propose a new model that does not assume electron donation, based on the following discussion.

### Photochromism with K desorption

**Figure 4:**
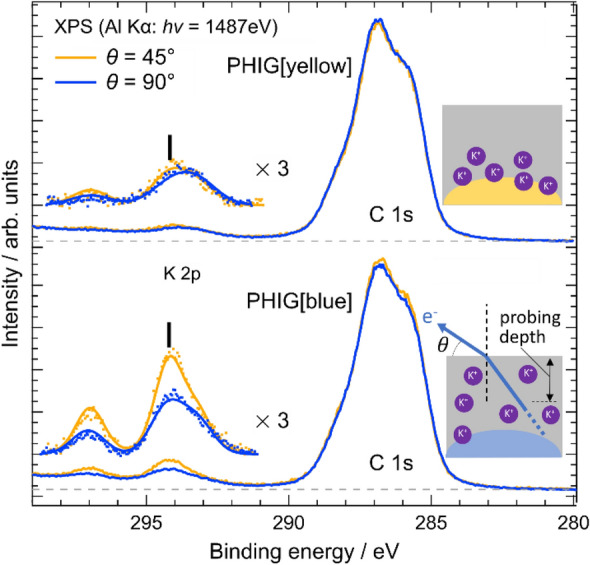
Angle-resolved XPS measurements of the C 1 s and K 2p core levels of PHIG[yellow] and PHIG[blue]. Colored dots are the measured spectra after subtracting Shirley-type backgrounds. Each spectrum was normalized by the intensity of the N 1 s peak of the Bmmim cation at around 402 eV. *θ* represents the take-off angle of the photoelectron from the sample surface, as illustrated in the figure.

Figure 4 shows the angle-resolved XPS spectra of PHIG measured at the take-off angle *θ* = 45° (yellow data and lines) and 90° (blue data and lines). The PHIG samples were obtained using [Bmmim]BF_4_. The upper and bottom panels show the results for PHIG[yellow] and PHIG[blue], respectively. The smaller the *θ*, the more sensitive the measurement at the sample surface. Table S1 shows the binding energies of K 2p and K/N_IL_ (the ratio of the amount of K to nitrogen contained in the Bmmim cation) in each sample. PHIG[yellow] shows only low intensity K 2p, regardless of the angle, whereas in PHIG[blue], the K 2p intensity was clearly angle dependent and increased at 45°. Given that the signal from the surface becomes stronger as Bmmim cation *θ* becomes smaller in XPS, the results in Fig. 4 show that there is more K^+^ on the surface of PHIG[blue], probably because the K contained in PHIG[yellow] was released into the ionic liquid as it changed to PHIG[blue]. Figure S5 shows the XPS results for [Bmmim]BF_4_, PHIG[yellow], PHIG[blue], and PHIG[blue] 90 min after light irradiation of PHIG[blue]. The intensity of K^+^ increases from PHIG[yellow] to PHIG[blue], and 90 min after terminating light irradiation, the intensity gradually decreases to its original value. These results suggest the involvement of K^+^ release in the photochromism of PHI.

**Figure 5:**
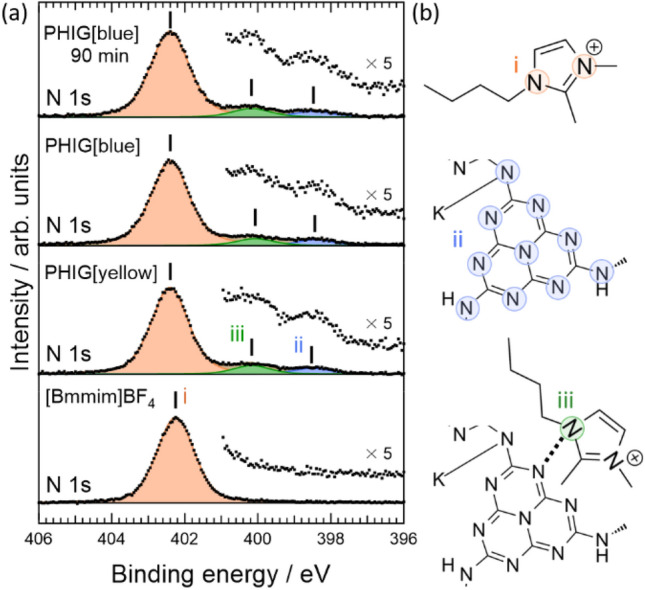
(**a**) XPS measurements of the N 1 s core level of [Bmmim]BF_4_, PHIG[yellow], and PHIG[blue]. The spectra for PHIG[blue] were acquired just after initiating light irradiation and 90 min after terminating irradiation. Shirley-type backgrounds were subtracted from the raw data. Each spectrum was normalized by the intensity of the N 1 s peak of the Bmmim cation at 402.25 eV. The labels i–iii on the spectra indicate the spectral features from nitrogen atoms in different environments, as illustrated in (**b**).

Next, we examined the chemical shifts of the N 1 s core levels because the release of K should alter the chemical environment of PHI. Figure 5a shows the N 1 s spectrum of the same samples as in Fig. 4. The labels i, ii, and iii in the figure indicate the contributions from different nitrogens, as illustrated in Fig. 5b: i and ii represent the nitrogen in the Bmmim cation and the one in PHI, respectively. Label iii represents the contribution from the nitrogen in the cation contacting the nitrogen in PHI through cation-π interaction. The results show that i barely shifts upon light irradiation, whereas ii and iii are shifted to 0.1 eV lower binding energy because ii is strongly bound to K if not irradiated with light. However, if K^+^ is released by light irradiation, ii is no longer affected by K and thus is the result of shifting the N 1 s core level to lower binding energy. Moreover, the nitrogen of iii was also affected by the shift of ii to lower binding energy. However, looking at Fig. S5, there is no significant shift of the K 2p core level because the K^+^ released from PHI is surrounded by BF_4_ anions and is immediately electrically neutralized. Note that iii is shifted in the same direction as ii rather than the opposite direction, indicating that no charge transfer occurs from [Bmmim]BF_4_ to PHI, consistent with the result that no charge transfer occurs between the electron donor and PHI in the photochromism of PHIG, as suggested in Fig. 3. In addition, since the energy gap of the ionic liquid is wide and its lowest unoccupied states are farther away from the top of the valence band of PHI, indeed, there is little possibility of charge transfer^24^. These results directly confirmed the movement of K^+^ upon light irradiation, affecting the chemical state of PHI. Thus, the release of K^+^ caused by light irradiation is involved in the photochromism of PHI.

### Reaction at the color change of PHIG

**Figure 6:**
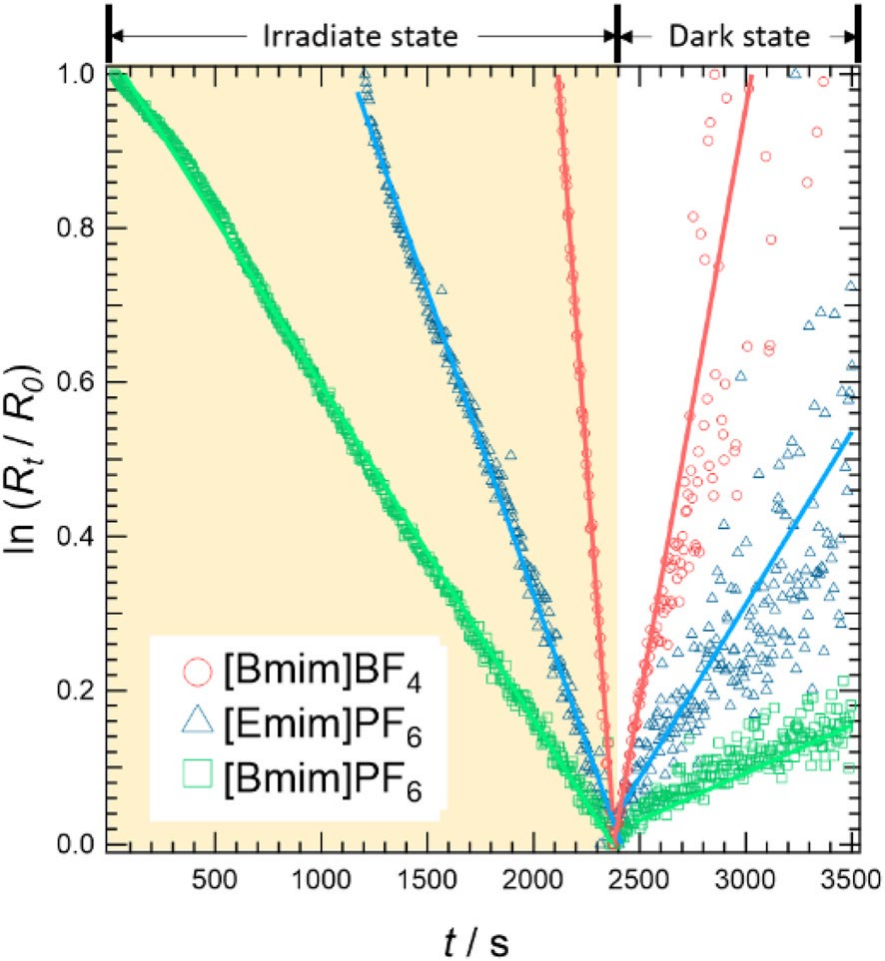
The time variation of the reflection spectrum intensity ln(*R*_*t*_/*R*_0_) at *λ* = 575 nm is shown. The bottom axis indicates the time *t* from the start of light irradiation and measurements. Symbols and solid lines indicate raw data and fitting lines, respectively. The “irradiated state” is the period during which the sample was continuously irradiated with white light, and the “dark state” is the period after irradiation was turned off.

Figure 6 shows the results of the time evolution of the reflection spectrum *R*_*t*_ of PHIG, where *t* is the time elapsed since the start of light irradiation. *R*_*t*_ was acquired at *λ* = 575 nm. The natural logarithm of the ratio *R*_*t*_/*R*_0_ was plotted against *t*, where *R*_*t*_ represents the reflected intensity at a certain time *t* and *R*_0_ represents the intensity at *t* = 0 s. As shown in Fig. 2, PHIG[blue] absorbs in the long wavelength region when irradiated by white light, so *ln*(*R*_*t*_/*R*_0_), which represents the reflection spectrum intensity, decreases during irradiation. *ln*(*R*_*t*_/*R*_0_) starts to return to the value for PHIG[yellow] when irradiation is stopped, so *ln*(*R*_*t*_/*R*_0_) starts to increase gradually. The color of PHIG changes slowly when the light is switched on. This exponential shift in the concentration of PHIG[blue] shown in Fig. 6 indicates that PHIG is in chemical equilibrium between PHIG[yellow] and PHIG[blue] in the dark state, that light irradiation triggers the system towards a new equilibrium position, and relaxation to the original equilibrium position occurs when irradiation is terminated. This transfer between two equilibrium states is described as follows:

1 $$\mathrm{PHIG} \; \left[\mathrm{yellow}\right]\underset{{k}_{b}}{\stackrel{{k}_{a}}{\rightleftarrows }}\mathrm{PHIG} \; \left[\mathrm{blue}\right] \;\; (\text{dark} \; \text{state})$$

2 $$\mathrm{PHIG} \; \left[\mathrm{yellow}\right]\underset{{k}_{b}^{^{\prime}}}{\stackrel{{k}_{a}^{^{\prime}}}{\rightleftarrows }}\mathrm{PHIG} \; \left[\mathrm{blue}\right] \; \left(\text{irradiation} \; \text{state}\right)$$

here, *k*_a_, *k*_b_, *k*_a_′, and *k*_b_′ are the rate constants for each reaction. The time evolution of change in the concentration of PHIG[blue] during relaxation from the dark state to the irradiated state is expressed by the following formula:

3 $$-\frac{{R}_{t}}{{R}_{0}}=\frac{{\left[\mathrm{PHIG} \; \left[\mathrm{blue}\right]\right]}_{t}}{{\left[\mathrm{PHIG} \; \left[\mathrm{blue}\right]\right]}_{0}}=\left(1-X\right){e}^{-{k}^{\mathrm{^{\prime}}}t}+X$$

where *k*′ means *k*_a_′ + *k*_b_′, and *X* is the ratio of [PHIG[blue]]_∞_ at *t* = ∞ s to [PHIG[blue]]_0_ at *t* = 0 s: [PHIG[blue]]_∞_ = *X* [PHIG[blue]]_0_. Given that the increase in [PHIG[blue]]_*t*_/[PHIG[blue]]_0_ represents an increase in the absorption of PHIG[blue], the reflection spectral intensity is equal to (− *R*_*t*_⁄*R*_0_). For each ionic liquid, *k* in the dark state is smaller than *k*′ in the irradiated state because in the dark state, the recovery of PHIG[yellow] → PHIG[blue] occurs relatively slowly, so *k*_b_ becomes smaller and the value of *k* is also smaller.

In addition, Fig. 6 shows that the slope of *ln*(*R*_*t*_/*R*_0_) changes for each ionic liquid. Interpreting these results requires consideration of the differences in the viscosity *η* of ionic liquids. The relationship between the viscosity of each ionic liquid is studied here [Bmim]PF_6_ > [Emim]PF_6_ > [Bmim]BF_4_^25^. Figure 6 shows that the slope of *ln*(*R*_*t*_/*R*_0_) decreases as *η* increases, indicating that *k* ∝ 1/*η*. The relationship between *η* and ionic diffusivity *D* in ionic liquids was reported by Noda et al*.* to be understood according to the Stokes–Einstein form, *D* ∝ 1/*η*^26^. In other words, the relation *k* ∝ *D* ∝ 1/*η* shows that the rate of change of PHIG, *k*, is directly correlated with the ion diffusion coefficient, *D,* through the viscosity of the ionic liquid used to form PHIG.

The results of Fig. 6 thus indicate that the change in PHIG is mediated by ion diffusion through the ionic liquid, consistent with the result that K^+^ ions are involved in this change, as discussed in Fig. 4. The dynamics of the color change in PHIG can thus be interpreted as follows. First, PHIG is in equilibrium between PHIG[yellow] and PHIG[blue], and this reaction is caused by the adsorption and desorption of K^+^ ions. During the change from PHIG[yellow] to PHIG[blue], K^+^ ions desorb from PHI, and in the reverse reaction, K^+^ ions adsorb onto PHI. Furthermore, as PHIG shifts to a new equilibrium state upon light irradiation, the ratio of PHIG[blue] to PHIG[yellow] changes and the ratio of PHIG[blue] becomes larger, so the color of PHIG appears blue. The higher the viscosity of PHIG, the more difficult it is for ions to diffuse, and thus K^+^ ions are less likely to desorb from PHI and take longer to recombine with PHI, resulting in the rate of change of PHIG being dependent on the viscosity. In the next section, how K adsorption/desorption affects the electronic structure of PHI will be discussed from a theoretical perspective to examine the mechanism underlying photochromism of PHI proposed above.

### Mechanism of the photochromism of PHI

**Figure 7:**
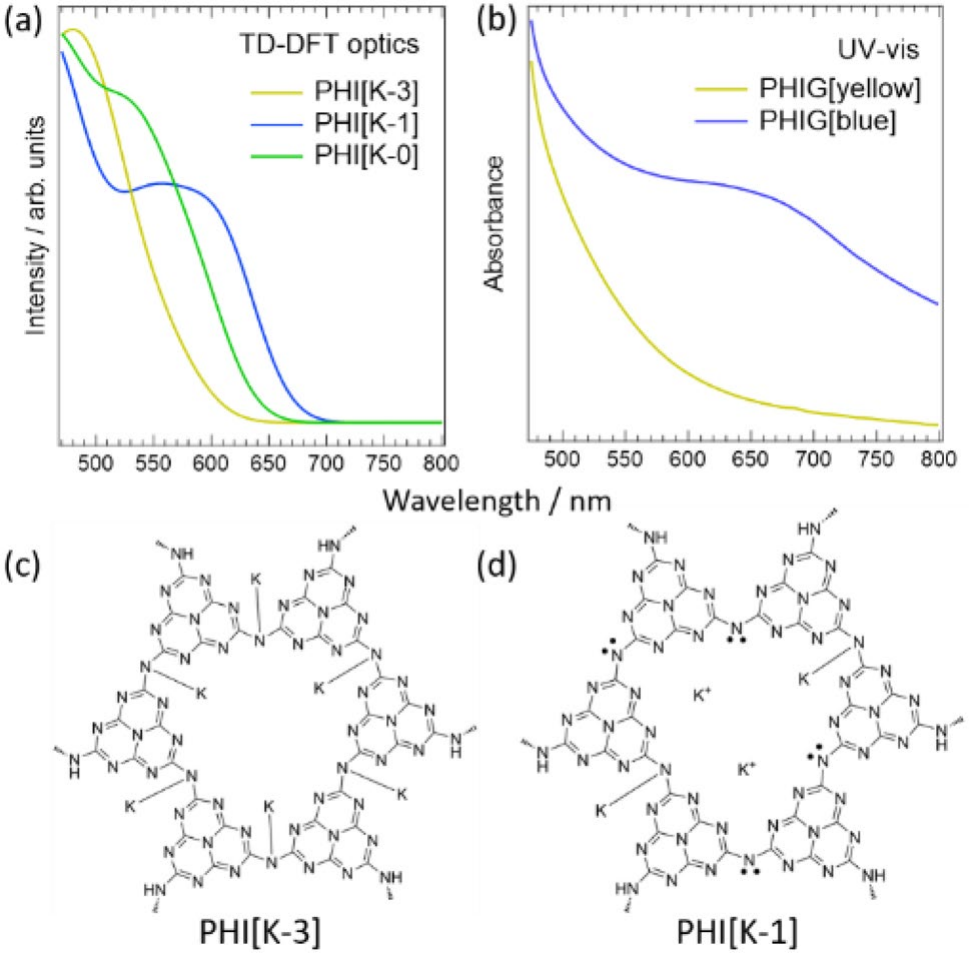
(**a**) Optical absorption spectra simulated by TD-DFT for the two PHI structures shown in (**c**) and (**d**). PHI[K-*n*] represents the structure of PHI in which *n* Ks are bound to the nitrogens in PHI. (**b**) Experimental UV–Vis spectra of PHIG[yellow] and PHIG[blue], previously shown in Fig. 2.

The above discussion suggests that K adsorption and desorption are involved in PHIG photochromism. This was investigated by TD-DFT. Figure 7a shows the results of the calculation of optical absorption for PHI[K-*n*] (*n* = 0, 1, and 3) using TD-DFT to be compared with the experimental UV–vis spectra of PHIG[yellow] and PHIG[blue] in Fig. 7b. A crystalline structure of PHI reported by Schlomberg et al. was used^19^ and the model structures for the calculation are shown in Fig. 7c,d.

The number of K–N bonds in one heptazine imide ring was denoted as *n*, and the system with *n* K–N bonds is denoted as PHI[K-*n*] in the figures. Note that in the following discussion, the quantitative value of the absorption edge is not discussed, but rather the relative spectral shape between the models and the experimental results are compared, in consideration of the effect of underestimation of the band gap calculated using DFT. Although the UV–Vis spectra of all the models shown in Fig. 7a have a large peak around 475 nm, PHI[K-1] and PHI[K-0] have a structure with a shoulder in addition to this band, whereas PHI[K-3] shows no shoulder structure. The shoulder structures observed in the results for PHI[K-1] and PHI[K-0] are also seen in the UV–Vis spectrum of PHI[blue], whereas PHIG[yellow] lacks this structure, as shown in Fig. 7b. This suggests that PHI[K-3] can be attributed as PHIG[yellow]. The spectrum of PHIG[blue] in Fig. 7b shows that the shoulder structure appears due to new absorption around 650 nm. In contrast, PHI[K-0] shows almost no absorption around 650 nm, whereas PHI[K-1] shows large absorption. Therefore, PHIG[blue] is considered to have a structure similar to PHI[K-1].

To support the model discussed above, we examined the chemical shifts of the N 1 s core level observed in the XPS results in Fig. 5a using DFT calculations. The calculated N 1 s core levels of PHI[K-3] and PHI[K-1] are summarized in Table S3. The core level of PHI[K-1] is 0.2 eV lower than the core level of PHI[K-3]. Furthermore, the XPS results show that the core level of PHIG[blue] is shifted to lower binding energy by about 0.1 eV, supporting the desorption of K in the photochromism mechanism of PHI discussed in Fig. 5a, further indicating that the core level of the nitrogen strongly bound to K shifts to lower binding energy upon its desorption.

**Figure 8:**
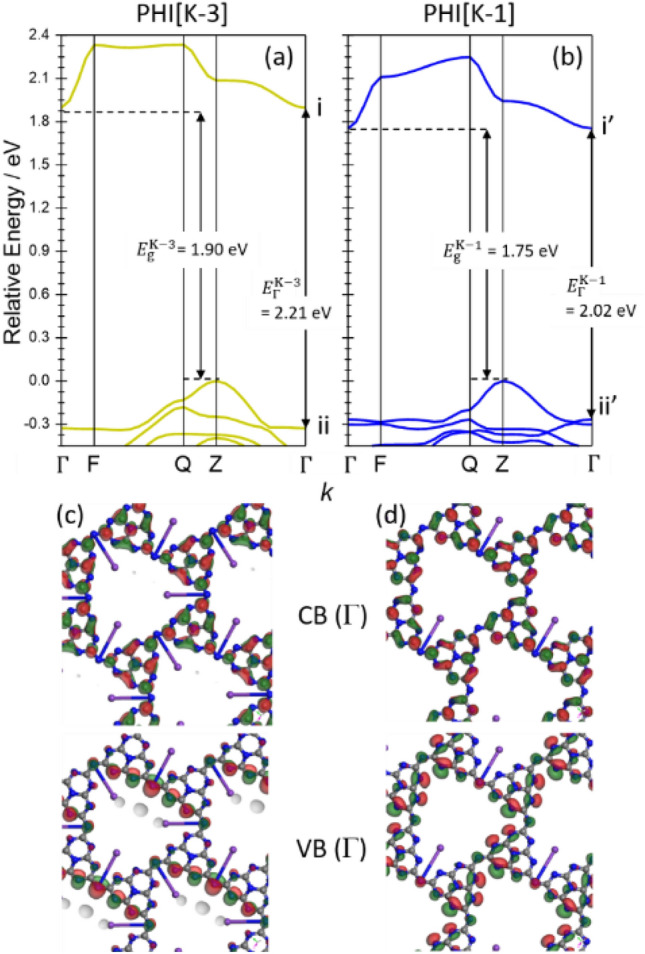
Energy band structures of PHI[K-3] (**a**) and PHI[K-1] (**b**) calculated by DFT. The left axes represent the energy measured from the top of the valence band. The letters on the bottom axes correspond to the letters in the Brillouin zones in Fig. S6c,d. Orbitals for valence band (VB) and conduction band (CB) at the Γ point of PHI[K-3] and PHI[K-1] are shown in (**c**) and (**d**), respectively. The green and red parts represent the lobe of the wavefunctions with opposite signs.

Based on the above discussion, it is reasonable to assume that PHI[K-1] is PHIG[blue] and PHI[K-3] is PHIG[yellow], which justifies the hypothesis that the band gap decreases with K desorption, causing photochromism, as discussed at Figs. 4 and 5. The interpretation that PHIG[blue] adopts primarily PHI[K-1] states rather than PHI[K-0] states can be understood by considering the entropy contribution to the Gibbs free energy of the system. Figure 8a,b shows the results of energy band calculations for PHIG[K-3] and PHIG[K-1], respectively. Figure 8c,d shows the valence and conduction band orbitals of PHI[K-3] and PHI[K-1] at the Γ point, respectively. Because the calculation of optical absorption shown in Fig. 7a was performed as a direct excitation at the Γ point, we will first discuss the energy gap *E*_Γ_ at the Γ point. The *E*_Γ_ of PHIG[K-1] is about 0.2 eV smaller than that of PHIG[K-3] and can be explained as follows. First, the shift of the upper edge of the valence band at the Γ point in PHIG[K-1] compared to PHIG[K-3] is due to the reduced symmetry of the system, increasing the degeneracy of the upper band of the valence states. Second, the lowering of the conduction band edge at the Γ point is attributed to the reduced contribution of nitrogen to the conduction band, as shown in Fig. 8c,d, where the contribution of nitrogen at the center of the heterocyclic ring almost disappears in PHI[K-1] compared to PHI[K-3]. Indeed, the calculated contribution of each element to the conduction band shows that the ratio of nitrogen to carbon is reduced by about 3%. Nitrogen is negatively charged due to its lone pair, lowering the orbital energy. Therefore, the conduction band with a reduced nitrogen contribution shifts to higher binding energy. Figure 8a,b shows that both PHI[K-3] and PHI[K-1] have an indirect gap, *E*g, indicating that the probability of charge recombination is low in PHI, i.e., charge separation is high. Indeed, as reported previously^11,27^, the photoluminescence intensity of PHI is lower than that of melon, which clearly indicates that PHI has high charge separation.

The discussion so far supports that the photochromism in PHIG is caused by K adsorption/desorption, and that the color change is caused by the change in the size of the energy gap as the structure of PHI changes.

### Mechanism of photoconductivity and dark photocatalysis of PHIG

**Figure 9:**
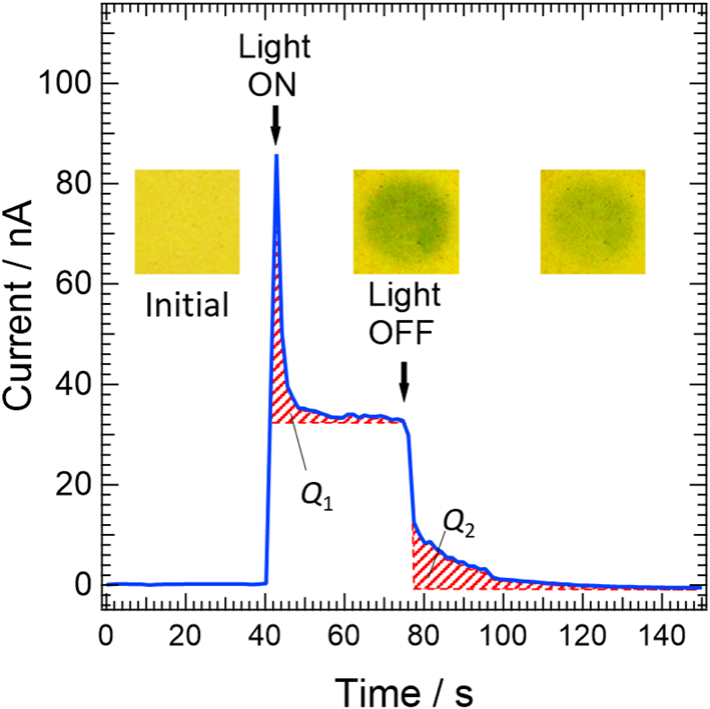
Time evolution of photocurrent in PHIG with a constant 0.5 V bias. “Light ON” represents the time when irradiation with white light was started and “Light OFF” represents the time when irradiation with white light was turned off. The inset photos are of PHIG taken under the same measurement conditions but the photo on the right was taken about 30 min after irradiation was terminated. Ionic liquid [Bmim]Br was used to obtain PHIG.

Figure 9 shows the time evolution of the photocurrent in PHIG with a 0.5 V bias. The photocurrent of PHIG shows a characteristic behavior after the light irradiation. PHIG gets in a low electric resistance state under white light irradiation due to the change from PHIG[yellow] to PHIG[blue] and the generation of photo-carriers. Importantly, the amount of charge represented by the areas labeled *Q*_1_ and *Q*_2_ shown in the figure are different: *Q*_2_ is larger than *Q*_1_. To clarify this discrepancy between *Q*_1_ and *Q*_2_, we divided the time variation of the photocurrent into four regions: (1) the region showing a sharp increase in photocurrent immediately after the light is turned on (within about 2 s); (2) the region where photocurrent sharply decreases immediately after (1) and then gradually decays (over about 30 s); (3) the region showing a rapid decrease (within about 1 s) after turning the light off; and (4) the region of slow decay with time (over 60 s after (3)). In (4), the photocurrent shows a long tail which lasts beyond 140 s.

Interpreting the photocurrents in Fig. 9 is aided by the discussion on photocurrents in organic solar cells by McNeil et al.^28^. First, in (1), a low resistance state occurs because photocarriers are generated in PHI immediately after the start of irradiation with white light. However, after entering region (2), the photocurrent peaks sharply and then immediately declines sharply because the generated photocarriers are trapped in PHI. After the sharp decline, photocarriers are gradually trapped, and the photocurrent slowly decreases. Consequently, the area shown in red mesh indicated by *Q*_1_ in Fig. 9 represents the total amount of trapped charge in PHI throughout region (2). In region (3), when irradiation is terminated, the photocarriers in PHI are expelled and the current decreases rapidly. In addition, the trapped carriers in PHI begin to be released in response to the electric field. Then, in region (4), the charge *Q*_1_ trapped in (2) is released gradually, and the current slowly decays. In this interpretation, the amount of charge *Q*_1_ trapped in (2) should correspond to the area of the red meshed area in (4) *Q*_2_, which represents the amount of charge released from the trap. Thus, in general, *Q*_2_ is never larger than *Q*_1_ and satisfies *Q*_1_ ≥ *Q*_2_ because there are basically two subsequent behaviors of a trapped charge: either it recombines within the trap, or it is released with a certain probability.

**Figure 10:**
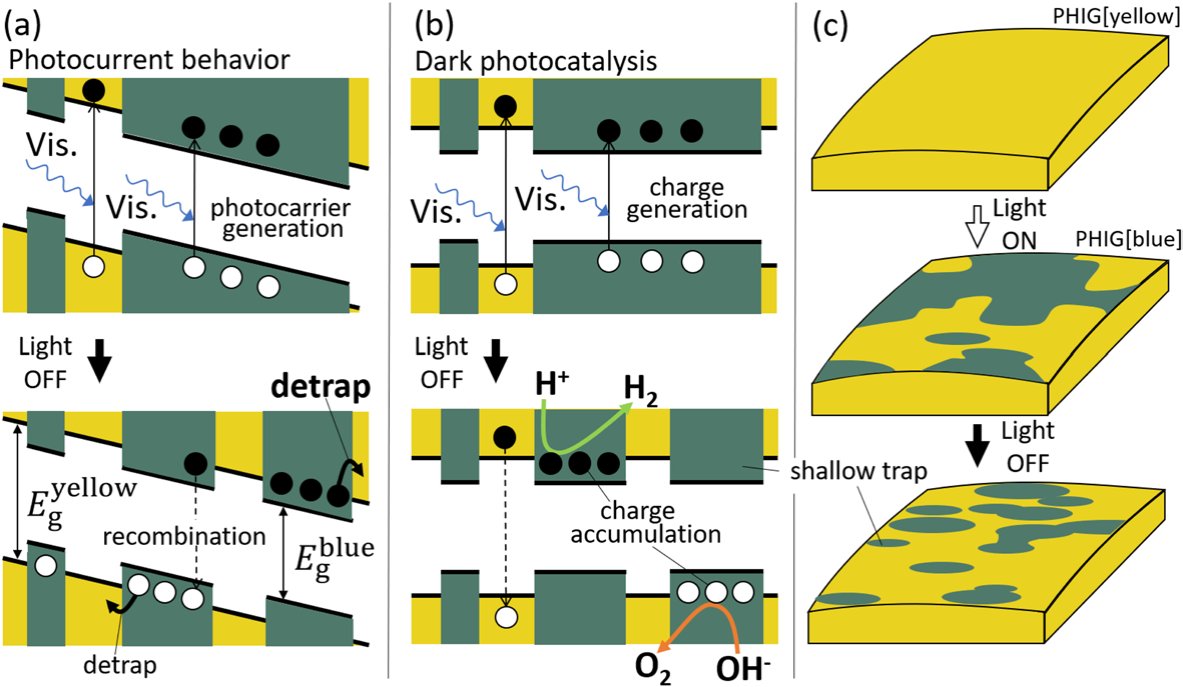
The shallow trap charge accumulation (STCA) model of PHIG. Upper parts of (**a**) and (**b**) show the energy diagrams of PHIG under light irradiation. The bottom parts of (**a**) and (**b**) show the energy diagrams of PHIG in the dark after stopping light irradiation. (**c**) Schematic of the change in the appearance of PHIG after light irradiation and after terminating irradiation. (**a**) Energy diagrams under a constant applied voltage, explaining the behavior of the photocurrent in Fig. 8. (**b**) Energy diagrams explaining dark photocatalysis by PHI. In the dark state after turning the light off, a portion of the carriers is left behind in the PHIG[blue] areas because the connections between the PHIG[blue] areas are severed. As the PHIG[blue] areas shrink, they become shallow charge traps, leading to increasing trapped charge, and some of the charge accumulates in the PHIG[blue] areas in PHIG.

The results of photocurrent measurements for melon gel are shown in Fig. S7. The gel was prepared in the same way as PHIG, using the same ionic liquid and melon, and *Q*_1_ > *Q*_2_ is satisfied. However, PHIG gives *Q*_1_ < *Q*_2_, which is inconsistent with the above results. It is usually not possible for the amount of charge released to be greater than the amount of charge trapped. Therefore, we consider this phenomenon to be peculiar to PHIG and propose the “shallow trap charge accumulation” (STCA) model to describe the behavior of carriers in PHIG, as shown in Fig. 10.

Figure 10a shows a conceptual energy diagram that explains the photocurrent shown in Fig. 9. The unusual situation *Q*_1_ < *Q*_2_ is considered in Fig. 10a. The upper part of Fig. 10a shows the situation where PHIG is irradiated with white light, and the state where optical carriers are generated in the PHIG[yellow] and PHIG[blue] regions. As can be seen from the photographs in Figs. 3 and 9, and as discussed in Fig. 6, not all the areas of PHIG irradiated with white light turn blue and a small number of yellow areas remain. The top and middle panels of Fig. 10c illustrate the changes in PHIG when exposed to light. As discussed in Fig. 6, PHIG is in an equilibrium state between PHIG[yellow] and PHIG[blue], so not all PHIG[yellow] becomes PHIG[blue] when exposed to light. PHIG[blue] has a smaller energy gap than PHIG[yellow], as discussed in Fig. 7, so PHIG[blue] acts as a shallow trap for the whole of PHIG.

Therefore, in (ii) in Fig. 9, some carriers are confined in PHIG[blue], and their total amount corresponds to the trapped charge *Q*_1_. When irradiation is stopped, the change from PHIG[blue] to PHIG[yellow] occurs actively, so the areas of PHIG[blue] gradually become smaller. As shown in Fig. 10c, the areas of PHIG[blue] appear as islands embedded in a sea of PHIG[yellow] and they become smaller with time. The resulting change in the appearance of PHIG can be seen in the photographs on the middle and right in Fig. 9. Importantly, the areas of PHIG[blue] that did not previously act as traps become new traps upon isolation from blue areas. In addition, the optical carriers that were supposed to leave from PHIG are trapped in the newly created trap of the PHIG[blue] islands. These newly trapped carriers cause *Q*_1_ < *Q*_2_, and in typical compounds such as melon, new traps are not generated when light irradiation is stopped, so *Q*_1_ ≥ *Q*_2_ is satisfied. The shallow traps created by PHIG[blue] islands are different from traps due to defects and impurities in other organic crystals. The charges that are trapped and localized in typical traps immediately recombine within a molecule and thus have a short exciton lifetime, whereas PHI has an indirect energy gap, as described in Fig. 8, and thus the charges trapped in PHIG[blue] islands have a low recombination probability. This low recombination probability appears in the rate of decrease of current observed in region (iv) in Fig. 9^18^. Indeed, as can be seen in the inset of Fig. S7, the rate of decrease in current when light irradiation is stopped is significantly smaller in PHIG than in melon gel. This is due to the low probability of carrier recombination because of the indirect gap in PHI and the short exciton lifetime due to the direct gap in melon, as reported by Akaike et al*.*^1^. Also, the carriers originally captured in the trap during irradiation and then released are captured again.

The time evolution of the photocurrent can thus be explained by the STCA model, and this model is also useful for understanding the mechanism of dark photocatalysis by PHI. Figure 10b shows conceptual energy diagrams to explain dark photocatalysis by PHI. Unlike in Fig. 10a, no voltage was applied to PHI and thus it is not necessary to consider the drift motion of the carriers in PHI. In the upper part of Fig. 10b, excitons generated by indirect transition by white-light irradiation are easily separated into electrons and holes. Turning off irradiation results in the state shown at the bottom of Fig. 10b and the charges separated in PHIG[blue] are confined in PHIG[blue] areas through the process described by the STCA model. These accumulated electrons and holes in the PHIG[blue] areas explain the photocatalytic activity of PHI even in the dark (after terminating light irradiation) because some of the accumulated charge undergoes a photocatalytic reaction without deactivation itself in the dark due to the low recombination probability of PHI. These insights provide hints for the development of new photocatalytic materials.

## Conclusion

PHIG, a novel substance obtained by the gelation of PHI with ionic liquids, allowed us to experimentally investigate interesting properties of PHI.

The change in color of PHI by visible light irradiation, and the dark photocatalysis by PHI reported previously, is explained by (1) photochromism and (2) charge accumulation, and they are strongly correlated. Combining XPS and reflection spectroscopy with DFT simulations showed that photochromism is caused by a change in the molecular structure of PHI associated with the adsorption/desorption of K. Interpretation of the time variation of the photocurrent of PHIG resulted in a model named STCA, which was proposed to clarify how charge accumulation occurs: by the capture of charges in regions where the electronic state is modified by light irradiation. In dark photocatalysis, these accumulated charges in PHI are used for the catalysis reaction.

The present findings using photo-stimuli responsive gels, and the mechanism of dark photocatalysis by PHI, provide new guidelines for the future development of new photocatalysts and photo-functional materials.

## Supplementary Information

Supplementary Information.
