# Supplementary material for: Mechanism of charge accumulation of poly(heptazine imide) gel
Source: Sci Rep. 2021 Sep 8;11:17833. doi: 10.1038/s41598-021-97025-9 (PMC8426363; doi:10.1038/s41598-021-97025-9)
Supplement: Supplementary file 1 — Supplementary Information. [file 41598_2021_97025_MOESM1_ESM.pdf]

Supporting Information

# Mechanism of Charge Accumulation of Poly(heptazine imide) Gel

## S1. FT-IR results of PHI and melon

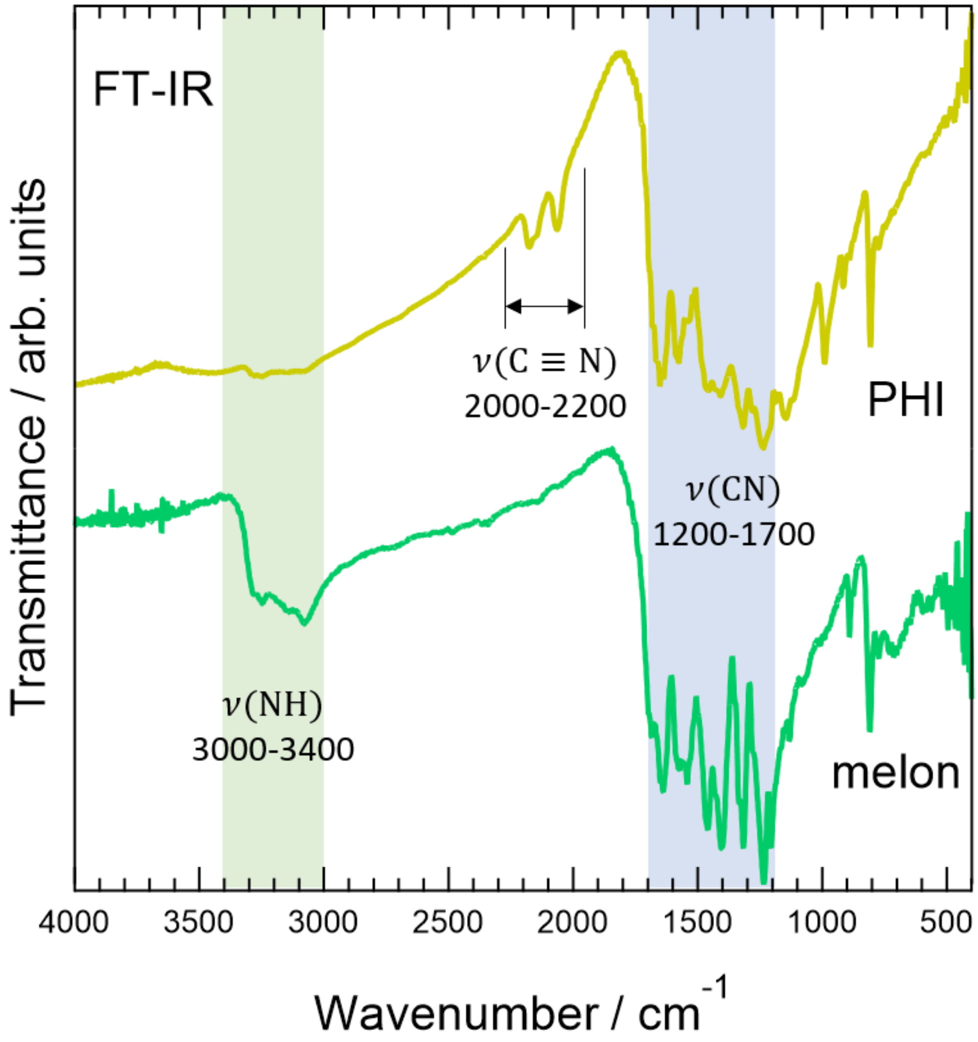


**Figure S1.** FT-IR spectra of powdery PHI and melon. The KBr pellet method was used to record the Fourier transform infrared (FT-IR) spectra. In the spectrum of PHI, the broad background at high wavenumbers is believed to be due the strong absorption of residual water used to wash the samples.

**S2. UV-vis results of PHI powder and PHIG**

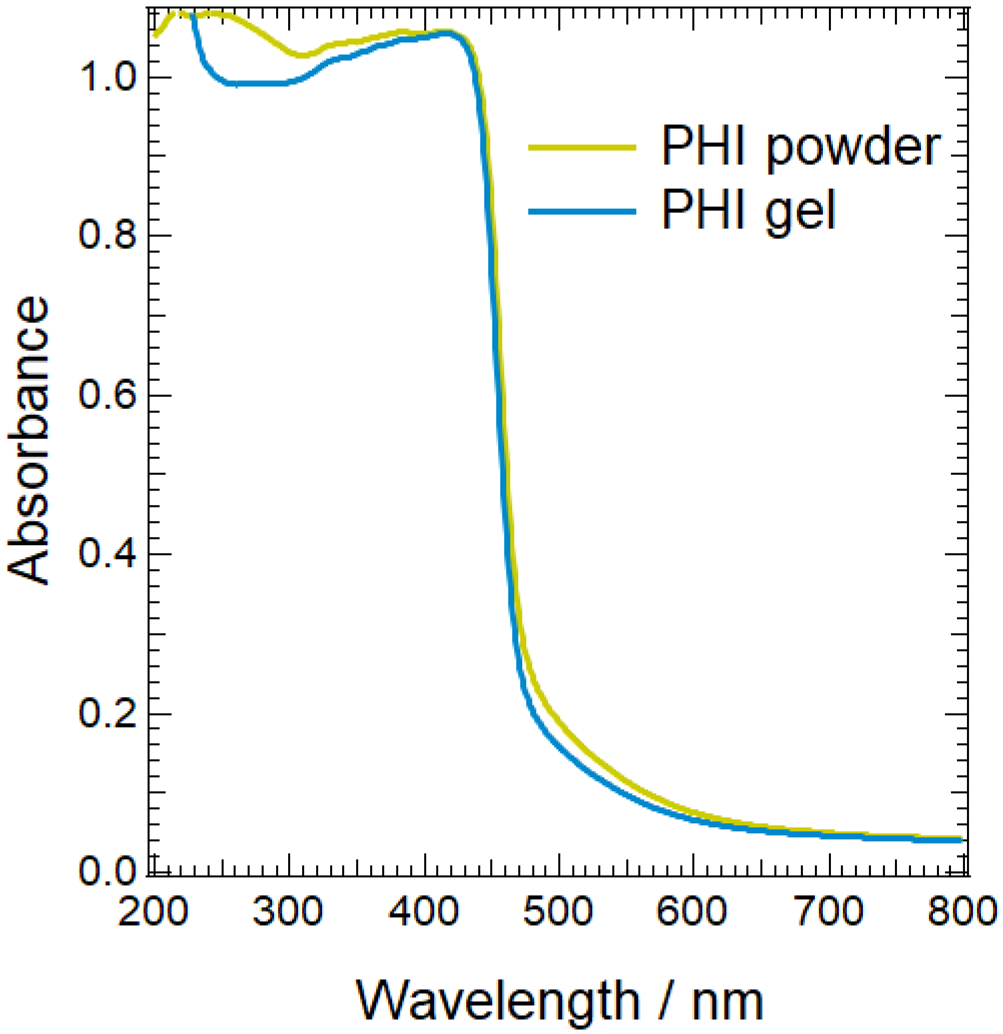


**FigureS2.** UV-vis spectra of PHI powder and PHI gel. In those spectrums, there isn't any difference between each spectrum. It means that PHI powder and PHI gel have same electronic structure, so that there is no degradation of PHI.

## S3. Color change of PHI with different organic electron donors and solvents.

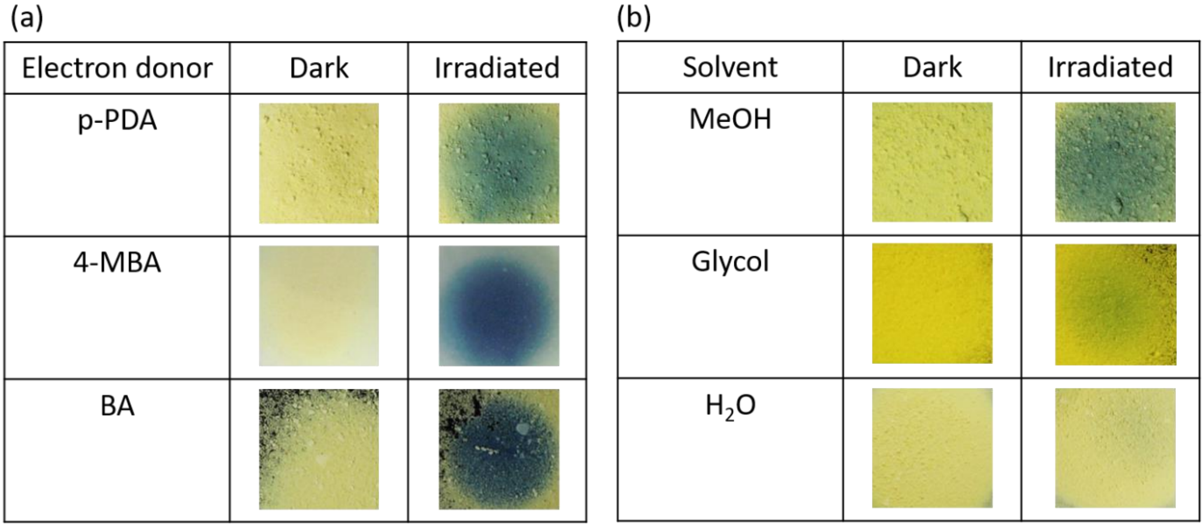


**Figure S3.** (a) Photographs of PHI + MeOH solutions with the organic electron donors p-PDA, 4-MBA and BA (each at 0.05 mol/L) before and after white-light irradiation. (b) Photographs of the PHI solutions prepared using the solvents MeOH, glycerin and $H_2O$ before and after white-light irradiation.

**S4. ESR measurements of PHI**

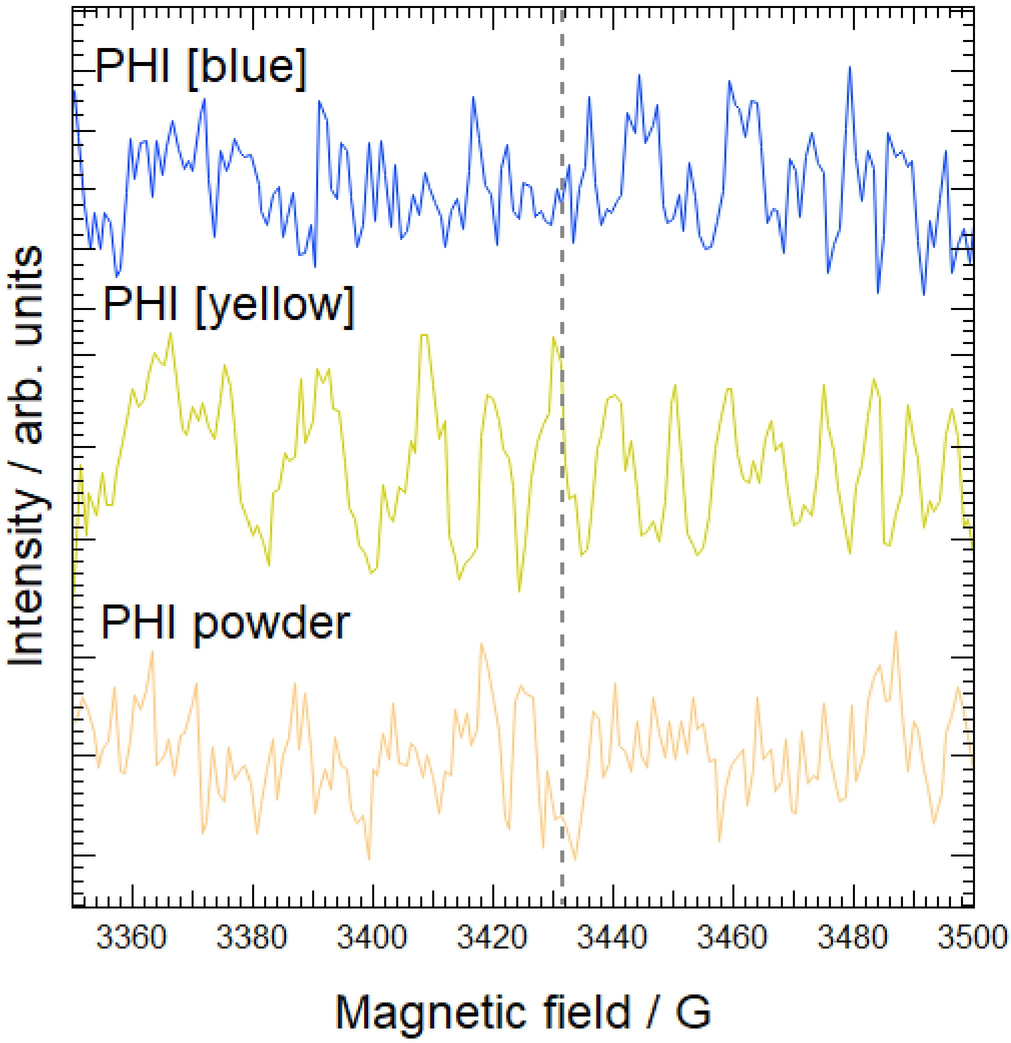


**Figure S4.** ESR results for a powdery sample of PHI and a MeOH solution of PHI, in which 4-MBA (0.05 mol/L) was mixed with a final concentration of 10% toluene to prevent dielectric loss. PHI[yellow] and PHI[blue] represent the samples before and after light irradiation. ESR measurements were performed using an E-55 ESR spectrometer. The parameters used were: signal gain = 20 dB, sweep time = 10 min, and modulation width = 5 $G_{p\text{-}p}$.

**S5. XPS measurements on K 2p core levels**

**Table S1**. The binding energy of the K $2p_{3/2}$ core level of PHIG[yellow] and PHIG[blue] observed at $\theta = 45°$ and 90°. The unit is eV. The numbers in brackets are the content ratio of K to $N_{IL}$. $N_{IL}$ means the nitrogen in the imidazolium cation in [Bmmim]$PF_4$, calculated from the XPS results.

| Sample | PHI [yellow] | PHI [blue] |
|---|---|---|
| 45° | 294.19 eV (2.63% ) | 294.20 eV (6.77%) |
| 90° | 294.17 eV (1.74%) | 294.37 eV (3.67%) |

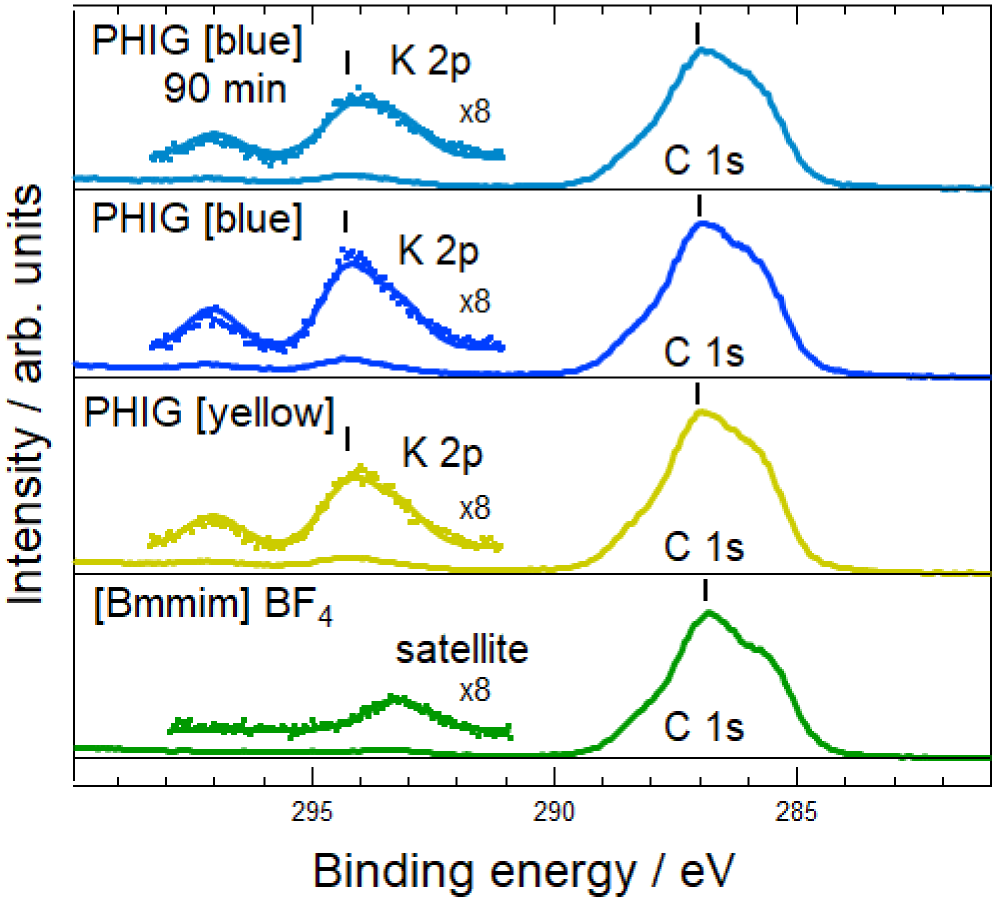


**Figure S5.** XPS measurements of the C 1s and K 2p core levels of PHIG[yellow], PHIG[blue] and [Bmmim]$PF_4$. Colored dots are the measured spectra after subtracting Shirley-type backgrounds. Each spectrum was normalized by the intensity of the C 1s peak of the Bmmim cation at around 287 eV. The spectrum labeled PHIG[blue] was acquired 90 min after stopping the irradiation of PHIG[blue]. The satellite observed in the spectrum of [Bmmim]$PF_4$ is a "shake-up satellite" caused by single electronic excitation of the Bmmim cation.

**Table S2.** The binding energy of the K $2p_{3/2}$ and N 1s core levels of $[Bmmim]BF_4$, PHIG[yellow], PHIG[blue], PHIG[blue] at 90 min. The unit is eV. The numbers in brackets are the content ratio of K to $N_{IL}$. $N_{IL}$ means the nitrogen in the imidazolium cation in $[Bmmim]PF_4$, calculated from the XPS results.

| **Sample** | **K2p** | **C1s** | **N1s** | |
|---|---|---|---|---|
| | | | $Bmmim^+$ | PHI |
| Bdim $BF_4$ | | 285.64eV (584%) | 402.25eV (100%) | |
| PHI [yellow] | 294.25eV (4.11%) | 285.83eV (480%) | 402.41eV (100%) | 398.53eV (6.96%) |
| PHI [blue] | 294.28eV (4.89%) | 285.81eV (415%) | 402.40eV (100%) | 398.44eV (6.83%) |
| PHI [blue] 90 min | 294.25eV (4.00%) | 285.84eV (433%) | 402.41eV (100%) | 398.48eV (7.06%) |

## S6. DFT calculation of the core level of each model

**Table S3.** The experimental binding energies of the K $2p_{3/2}$ and N 1s core levels of PHIG[yellow] and PHIG[blue]. The calculated core level energies of PHI[K-1] and PHI[K-3] are shown. For discussion of energy shifts, the energy values of PHI[K-3] are matched to the binding energy values of the N 1s and K 2p core levels of PHIG[yellow] determined experimentally by XPS and shown in Fig. 5.

| | **Sample** | **N 1s** | **K 2p** |
|---|---|---|---|
| XPS | PHIG [yellow] | 398.53eV | 294.25eV |
| | PHIG [blue] | 398.44eV | 294.28eV |
| | **model** | **N 1s** | **K 2p** |
| Core level calculation | PHI [K-3] | 398.53eV | 294.25eV |
| | PHI [K-1] | 398.33eV | 294.84eV |

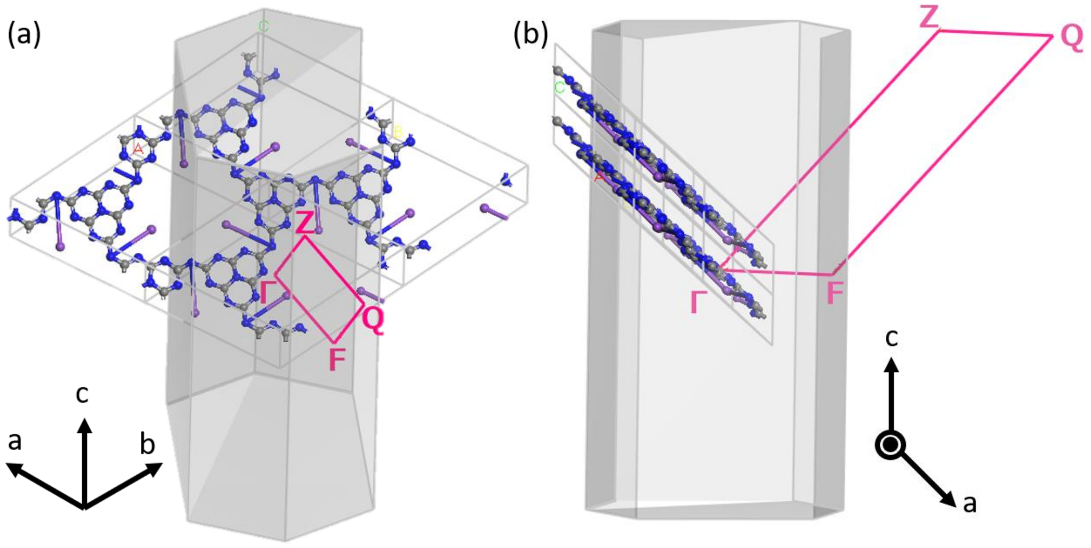


**Figure S6.** Brillouin zone of PHI[K-3]. Each k point is coincident to the energy band structure in Fig. 8(a) and (b). Note that PHI[K-1] has the same Brillouin zone as PHI[K-3] because it has the same crystal structure.

## S7. Photocurrent of melon gel

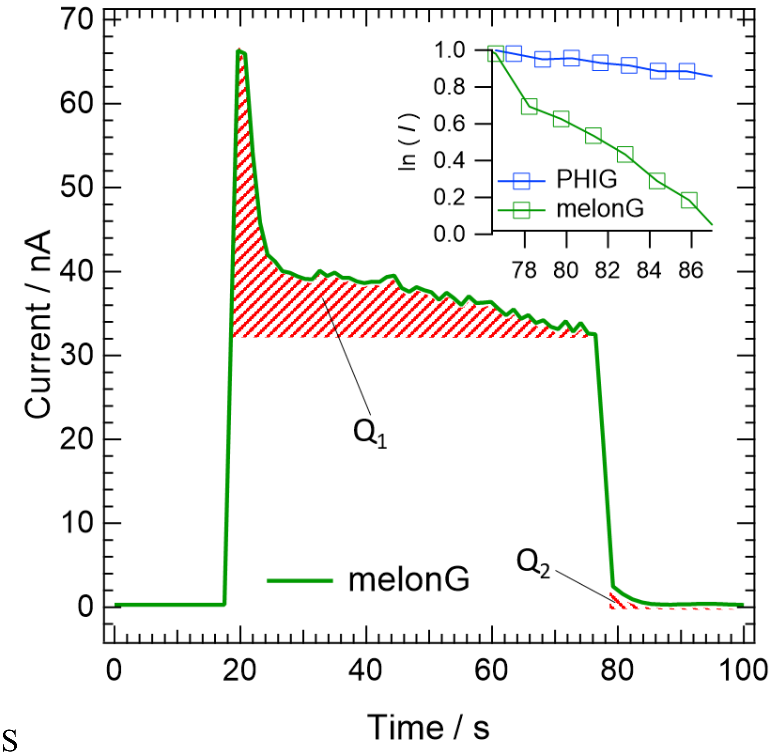


S

**Figure S7.** Photocurrent of melon gel (melonG) plotted against time. MelonG was prepared in the same manner as PHIG using the ionic liquid [Bmim]Br. The inset shows the photocurrent of PHI and melon after terminating irradiation with white light.
